# Application of artificial intelligence models for detecting the pterygium that requires surgical treatment based on anterior segment images

**DOI:** 10.3389/fnins.2022.1084118

**Published:** 2022-12-20

**Authors:** Fan Gan, Wan-Yun Chen, Hui Liu, Yu-Lin Zhong

**Affiliations:** ^1^Medical College of Nanchang University, Nanchang, China; ^2^Department of Ophthalmology, Jiangxi Provincial People’s Hospital, The First Affiliated Hospital of Nanchang Medical College, Nanchang, China

**Keywords:** anterior segment images, artificial intelligence, deep learning, pterygium, surgery

## Abstract

**Background and aim:**

A pterygium is a common ocular surface disease, which not only affects facial appearance but can also grow into the tissue layer, causing astigmatism and vision loss. In this study, an artificial intelligence model was developed for detecting the pterygium that requires surgical treatment. The model was designed using ensemble deep learning (DL).

**Methods:**

A total of 172 anterior segment images of pterygia were obtained from the Jiangxi Provincial People’s Hospital (China) between 2017 and 2022. They were divided by a senior ophthalmologist into the non-surgery group and the surgery group. An artificial intelligence model was then developed based on ensemble DL, which was integrated with four benchmark models: the Resnet18, Alexnet, Googlenet, and Vgg11 model, for detecting the pterygium that requires surgical treatment, and Grad-CAM was used to visualize the DL process. Finally, the performance of the ensemble DL model was compared with the classical Resnet18 model, Alexnet model, Googlenet model, and Vgg11 model.

**Results:**

The accuracy and area under the curve (AUC) of the ensemble DL model was higher than all of the other models. In the training set, the accuracy and AUC of the ensemble model was 94.20% and 0.978, respectively. In the testing set, the accuracy and AUC of the ensemble model was 94.12% and 0.980, respectively.

**Conclusion:**

This study indicates that this ensemble DL model, coupled with the anterior segment images in our study, might be an automated and cost-saving alternative for detection of the pterygia that require surgery.

## Introduction

Pterygium is a common ocular surface lesion characterized by wing-shaped, fibrovascular conjunctival outgrowth that invades the clear cornea (Xue et al., 2014). The pathogenesis of pterygia is still not completely understood, several factors including ultraviolet radiation, immunoinflammatory process, virus infection, and genetic factors have been reported to be related to pterygial formation (Chen et al., 2010). A pterygium not only affects facial appearance but can also grow into the tissue layer, causing astigmatism and vision loss. A pterygium is divided commonly into active and stationary phases. In stationary phase, it is relatively flat, the color is light red or white, congestion is not obvious, and growth is slow. In active period, it is thickened, congestion is obvious, the color is red, and growth is faster. Surgical treatment should be administered in a timely manner for an active pterygium to prevent damage to the ocular surface caused by cytokines from pterygium (Liu et al., 2020).

In a clinical setting, ocular surface diseases are diagnosed by professional ophthalmologists based on anterior segment images. In primary medical institutions, such as community hospital, there are few professional ophthalmologists, so it is difficult to diagnose a pterygium, let alone identify a pterygium that requires surgical treatment. This might result in a delay to surgical intervention, and the optimal treatment time being missed. Unfortunately, surgical removal of an advanced pterygium also carries higher risk of post-operative complications, such as corneal scarring, post-operative complication-induced astigmatism, higher rates of recurrence, and so poorer prognosis (Fang et al., 2021). Therefore, it is necessary to detect and refer pterygium requiring surgical treatment timely. For this reason, an automated approach for detecting a pterygium that requires surgical treatment is needed.

With the development of artificial intelligence (AI), it is being applied increasingly in various healthcare disciplines, especially in fields, such as ophthalmology, in which medical image assessment has a key role (Tang et al., 2022). DL algorithms have been used widely to diagnose fundus diseases such as diabetic retinopathy (DR), diabetic macular edema (DME), central serous retinopathy (CSR), and age-related macular degeneration (AMD) (Gulshan et al., 2016; Syed et al., 2016; Chen et al., 2021; Tang et al., 2021) and has good results. Few previous studies using AI to diagnose ocular surface diseases. Pterygium is one of the most common ocular surface diseases. Zaki et al. (2018) used support vector machine (SVM) to detect pterygium. This study on pterygium detection was based on the two-class detection of pterygium and traditional machine learning. It has not been further determined whether the pterygium requires surgical treatment. Hung et al. (2022) used a multi-layer perception (MLP) model to perform pterygium grading and further predict surgical prognosis. Zheng et al. (2021) used MobileNet, AlexNet, VGG16, and the ResNet18 model for pterygium grading. These studies all based on the traditional DL models. The performance of these models still needs to be improved.

Ensemble learning is a kind of machine-learning paradigm in which multiple models, such as decision trees, neural networks, and support vector machine (SVM), are combined together to solve a particular problem (Wang et al., 2015). An ensemble of various machine-learning models could help to reduce the bias in a single machine-learning algorithm to provide a much better prediction performance than single models (Kong et al., 2021).

Therefore, in this study, an ensemble scheme consisting of four DL model and multilayer perceptron (MLP) classifier was designed with aim to detect a pterygium that requires surgical treatment. In anticipation of detecting and referring pterygium requiring surgical treatment timely in the clinic work applying in treatment selection in personalized precision therapy of the pterygium. Thus, we hypothesized that an ensemble of various machine-learning models could help to provide a much better prediction performance than single models.

## Materials and methods

### Dataset preparation

**FIGURE 1:**
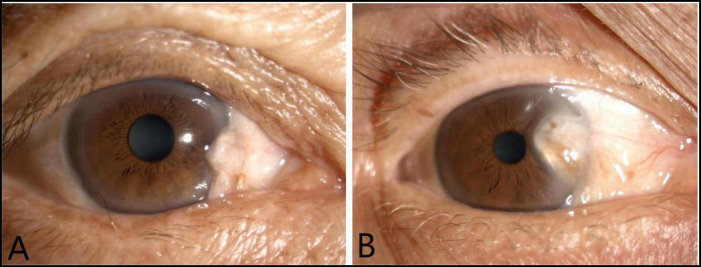
The anterior segment images of two group: The anterior segment image of the non-surgery group **(A)**. The anterior segment image of the surgery group **(B)**.

The total of 172 anterior segment images of pterygium were obtained from the Jiangxi Provincial People’s Hospital between 2017 and 2022. All the images selected had high quality and were obtained from the same slit lamp digital microscopy. They were divided by a senior ophthalmologist into the non-surgery group and the surgery group. The classifying standard was as follows (Zheng et al., 2021). The anterior segment images of the non-surgery group was characterized by the horizontal length of the pterygium head tissue invading the limbus of the cornea <3 mm. The anterior segment images of the surgery group was characterized by the horizontal length of the pterygium head tissue invading the limbus of the cornea ≥3 mm. LabelMe software was applied to label the regions of interest (ROIs) of the anterior segment images. The non-surgery group was labeled “label 0” and the surgery group was labeled “label 1.” To avoid over fitting problems, the dataset was established and divided by stratified sampling into training set (*n* = 136) and testing set (*n* = 34) at a ratio of 8:2 by referring to the previous research (Lu et al., 2022), ensuring that there was no overlap between the same person’s data in the development and internal test sets. The anterior segment images of two group were shown in Figure 1.

### Model development

**FIGURE 2:**
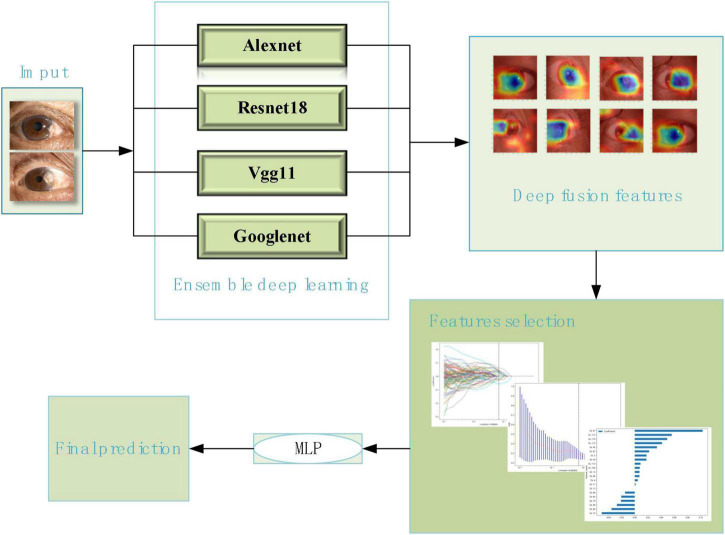
The flowchart of the ensemble deep learning model.

This study developed five prediction models based on deep learning neural network models, Which were resnet18 model, alexnet model, googlenet model, vgg11 model, and the ensemble deep learning (DL) model, respectively. The ensemble DL model was integrated with the alexnet, googlenet, vgg11, and resnet18 models. The flowchart of the ensemble DL model as shown in Figure 2.

During the training process of deep learning, the anterior segment images on the training set and the classification labels of pterygium was entered into the five deep learning models, separately. Convolutional neural networks (CNNs) coupled with batch normalization layers were used and the convolutional layer weights were initialized based on the ImageNet Large Scale Visual Recognition Challenge (ILSVRC) dataset pre-trained models (Fang et al., 2021). And then these CNNS were used to extract features. Usually, lower CNN layers are used to extract abstract features like edges, and deeper CNN layers are used to find features that are informative for the target task (Shen et al., 2017). Deep feature on “avgpool” layer before last FC layers were extracted. These extracted features from each model were screened by Least absolute shrinkage selection operator (LASSO) method separately. Finally, these selected features were then used to build classification model through a multilayer perceptron (MLP) neural network. The LASSO method and MLP neural network were also a part of deep learning models. And then the models were applied to the test set.

### Model visualization

The gradient-weighted class activation mapping (Grad-CAM) is a widely used method to interpret which features are responsible for determining outputs. It functions by capturing a specific class’s vital features from the last Conv layer of a CNN model to localize its important regions (Chen et al., 2010; Kwon et al., 2020; Montalbo, 2021).

To understand which areas of the anterior segment images of our dataset were most likely to be used by the model to predict whether the pterygium requires surgical treatment. We used Grad-CAM for visualizing the filters of the penultimate layer of the deep learning process, that can highlight which parts of an image contribute to the deep learning models.

### Statistical analysis

The accuracy, sensitivity, specificity, F1-score, confusion matrix and the area under the receiver operating characteristic curve (AUC) of the prediction models on the training set and test set were calculated, separately. And the predictive performances of the ensemble DL, resnet18, googlenet, alexnet, and vgg11 models were compared based on the above indicators.

### Software

The anterior segment images labeled using the LabelMe Open Annotation Tool.^1^ Deep learning techniques were completed using Python version 3.9.

## Results

A total of 172 anterior segment images of pterygia are in our dataset. Of these, 95 images were labeled as the surgery group and 77 images were labeled as the non-surgery group. The dataset was divided by stratified sampling into training and testing sets at a ratio of 8:2. The 136 anterior segment images of pterygia in the training set were used to train the ensemble DL model, Resnet18 model, Googlenet model, Alexnet model, and Vgg11 model. The 35 images in the testing set were used to test the models.

**FIGURE 3:**
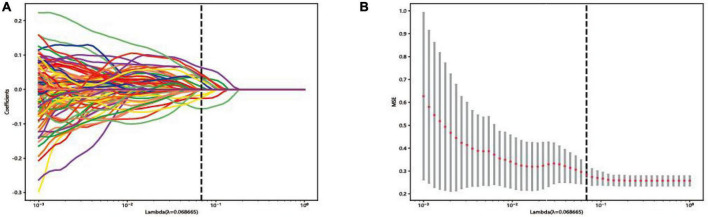
Feature selection in the least absolute shrinkage selection operator (LASSO) model: **(A)** LASSO coefficient profiles of the features. **(B)** Selection of tuning parameters in the Lasso regression analysis by 10-fold cross-validation.

The LASSO model was used to screen independent predicting features of ensemble DL model in training set. Each feature has a coefficient as its weight provided by LASSO, when the binomial deviance was minimized, features with non-zero coefficients were selected by optimal lambda. The optimal lambda was 0.068665, as shown in Figure 3.

Compared with other models, the ensemble DL model achieved the highest accuracy in both the training and testing sets. The accuracy was 94.20 and 94.12% in the training set and testing set, respectively. The accuracies of the Alexnet, Googlenet, Resnet18, and Vgg11 model in the training set were only 85.40, 84.67, 80.29, and 84.67%, respectively; whereas their accuracies were only 85.71, 80.00, 77.14, and 85.71% in the testing set. All results were shown in Table 1.

**TABLE 1:** The performances of the models of the models.

Models	Train/Test	Accuracy	AUC	95% CI	Sensitivity	Specificity	F1-score
Ensemble DL	Train	94.20%	0.978	(0.958–0.998)	90.79%	98.39%	94.52%
	Test	94.12%	0.980	(0.968–1.000)	89.47%	100.00%	94.44%
Alexnet	Train	85.40%	0.900	(0.847–0.953)	90.79%	78.69%	87.34%
	Test	85.71%	0.878	(0.754–1.000)	89.47%	81.25%	87.18%
Googlenet	Train	84.67%	0.912	(0.867–0.958)	77.63%	93.44%	84.89%
	Test	80.00%	0.872	(0.759–0.985)	68.42%	93.75%	78.79%
Resnet18	Train	80.29%	0.835	(0.765–0.906)	82.89%	77.05%	82.35%
	Test	77.14%	0.816	(0.673–0.959)	63.16%	93.75%	75.00%
Vgg11	Train	84.67%	0.920	(0.878–0.963)	85.52%	83.61%	86.10%
	Test	85.71%	0.921	(0.836–1.000)	94.74%	75.00%	87.80%

**FIGURE 4:**
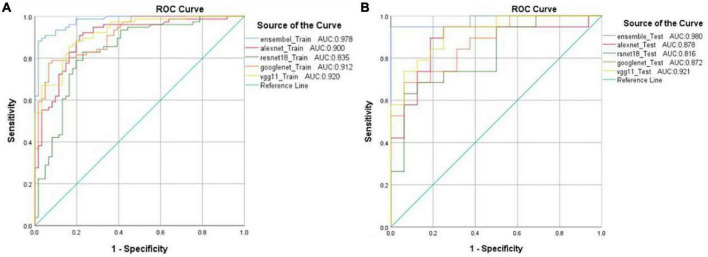
Receiver operating characteristic (ROC) curves and the area under the receiver operating characteristic (AUC) values from five different models in the training set **(A)** and testing set **(B)**.

All the models had good results with AUC (Table 1 and Figure 4), of which the ensemble DL model had the highest AUC in both the training and testing set, of 0.978 and 0.980, respectively. The AUC of the other models in the training and testing sets were all <0.950.

**FIGURE 5:**
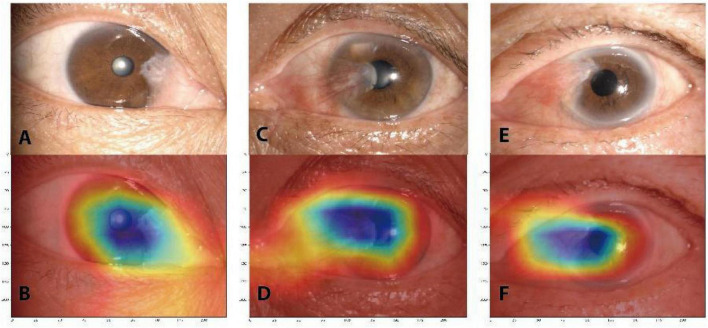
Gradient-weighted class activation mapping (Grad-CAM) heatmaps that highlight important regions for the deep learning model predicted for pterygium classification. The original anterior segment images **(A,C,E)**. The corresponding heatmaps **(B,D,F)**.

The heatmaps of Grad-CAM highlighted areas of the anterior segment images where the DL models probably focused on detecting a pterygium that required surgical treatment. The areas highlighted were the actual sites that correspond well with the pterygium (Figure 5).

## Discussion

In this study, we developed an AI model based on ensemble DL that was integrated with the alexnet, googlenet, vgg11, and resnet18 models for detecting the pterygium that requires surgical treatment and visualizes the DL process by Grad-CAM.

Compared with the classical Resnet18, Alexnet, Googlenet, and Vgg11 models, respectively, the results showed that the ensemble DL model achieved the highest accuracy and AUC than the four classical DL models in both the training and testing sets. The accuracy of the ensemble DL model was up to 94%. The maximum accuracy of the classical DL models was only 85%. The ensemble DL model outperformed classical DL models with an improvement in accuracy of 9%. The AUC of the ensemble DL model was up to 98% and was at least 6% higher than that of the other four classical models. Thus, our results indicated that this ensemble DL model coupled with the anterior segment images might be an automated and cost-saving alternative for detecting the pterygium that require surgery.

In the previous study, Zaki et al. (2018) used an artificial neural network (ANN) and a SVM to detect a pterygium. The average accuracy reached 91.27% and the AUC reached 95.6%. Hung et al. (2022) developed a DL system in pterygium grading based on the multilayer perceptron (MLP) and the accuracy was 86.67 to 91.67%. Xu et al. (2021) used MobileNet, AlexNet, VGG16, and ResNet18 models to diagnose pterygia. The highest accuracy of the models was 88.30% and the highest AUC of the models was 0.872. However, the accuracy and AUC of our ensemble DL model was up to 94 and 98%. In comparison, the performance of our model was better. It could be related to the method that they used, which was classical machine learning or deep learning method. Compared with the traditional method, DL is a mainly data-driven feature extraction, which does not require much feature extraction of specific domain knowledge, and can extract deep abstract features that are difficult to extract by the traditional method. Its expression of data sets is more efficient and accurate, and the extracted abstract features are more robust and have better generalization ability. Also, compared with the classical DL models, ensemble learning has the advantages of improving prediction performance, directly cascading different models, easy implementation, and fewer parameters.

Precision medicine is a medical model for prevention, diagnosis, and treatment that aims to achieve an optimal therapeutic regimen for an individual (Yan et al., 2021). It has become a focal area of interest and development in medicine of the 21st century (Iacobas and Xi, 2022). Previous researchers such as Zaki et al. (2018); Zulkifley et al. (2019), and Zamani et al. (2020) was based on the two-class detection of pterygium. Unlike their studies, the model of our study was developed to further detect the pterygium that requires surgical treatment. This more closely fits with the current strategy for precision medicine.

Another key strength of this study was the use of Grad-CAM. The disadvantage of ensemble learning is that the prediction results are as uninterpretable as the DL model, i.e., black-box system (Yamashita et al., 2021). Therefore, the Grad-CAM was introduced in our study for visualizing the filters of the penultimate layer of the DL process. The result of Grad-CAM heatmaps highlighted important regions for the DL model predicted for pterygium classification and these regions were consistent with the actual location of the pterygium. It indicated that the model was making predictions based on clinical features of the pterygium.

Of course, our study has some shortcomings. Our sample size was small and might limit the generalizability of our findings. Our study also did not combine clinical features in models. The majority of studies showed that imaging features combined with clinical features have a high value in predicting and diagnosing (Stubblefield et al., 2020; Zhou et al., 2021; Huang et al., 2022). In future research, we will continue to enroll more cases and use the data augmentations method to addresses issues of small sample sizes. And we will also combine with clinical features to detect pterygia that require surgery to improve the performance of the model.

## Conclusion

We developed an AI model based on ensemble DL method to classify pterygium. The results indicated that ensemble DL model based on the anterior segment images might be an automated and cost-saving alternative for detection of the pterygium that require surgery. We also used the Grad-CAM to visualize the DL process. The highlighted important regions in the Grad-CAM heatmaps were consistent with the actual location of the pterygium. It indicated that the model was making predictions based on clinical features of the pterygium.

## Data availability statement

The raw data supporting the conclusions of this article will be made available by the authors, without undue reservation.

## Ethics statement

The studies involving human participants were reviewed and approved by institutional review board of Jiangxi Provincial People’s Hospital. The patients/participants provided their written informed consent to participate in this study. Written informed consent was obtained from the individual(s) for the publication of any potentially identifiable images or data included in this article.

## Author contributions

FG, W-YC, HL, and Y-LZ: statistical analyses and wrote the manuscript. All authors read and approved the final manuscript, contributed to data collection and article, and approved the submitted version.

## Footnotes

1
http://labelme.csail.mit.edu
